# Transcriptional Profiling of *Staphylococcus aureus* during the Transition from Asymptomatic Nasal Colonization to Skin Colonization/Infection in Patients with Atopic Dermatitis

**DOI:** 10.3390/ijms25179165

**Published:** 2024-08-23

**Authors:** Peijuan Li, Julia Schulte, Gerda Wurpts, Mathias W. Hornef, Christiane Wolz, Amir S. Yazdi, Marc Burian

**Affiliations:** 1 Department of Dermatology and Allergology, RWTH University Hospital Aachen, D-5207 Aachen, Germany; pli@ukaachen.de (P.L.);; 2 Institute of Medical Microbiology, RWTH University Hospital Aachen, D-52074 Aachen, Germany; 3 Interfaculty Institute of Microbiology and Infection Medicine, University of Tuebingen, D-72076 Tuebingen, Germany; 4 Cluster of Excellence EXC 2124 “Controlling Microbes to Fight Infections”, University of Tuebingen, D-72076 Tuebingen, Germany

**Keywords:** in vivo gene expression, nasal colonization, human skin, skin habitat, accessory gene regulator (*agr*), proteases, infection, virulence regulator, adhesins

## Abstract

*Staphylococcus aureus* acts both as a colonizing commensal bacterium and invasive pathogen. Nasal colonization is associated with an increased risk of infection caused by the identical strain. In patients with atopic dermatitis (AD), the degree of *S. aureus* colonization is associated with the severity of the disease. Here, we comparatively analyzed the in vivo transcriptional profile of *S. aureus* colonizing the nose and non-diseased skin (non-lesional skin) as opposed to the diseased skin (lesional skin—defined here as infection) of 12 patients with AD. The transcriptional profile during the asymptomatic colonization of the nose closely resembled that of the lesional skin samples for many of the genes studied, with an elevated expression of the genes encoding adhesion-related proteins and proteases. In addition, the genes that modify and remodel the cell wall and encode proteins that facilitate immune evasion showed increased transcriptional activity. Notably, in a subgroup of patients, the global virulence regulator Agr (accessory gene regulator) and downstream target genes were inactive during nasal colonization but upregulated in the lesional and non-lesional skin samples. Taken together, our results demonstrate a colonization-like transcriptional profile on diseased skin and suggest a role for the peptide quorum sensing system Agr during the transition from asymptomatic nasal colonization to skin colonization/infection.

## 1. Introduction

The multifaceted interaction of *Staphylococcus aureus* with the human host is illustrated by its variety of clinical presentations, ranging from harmless colonization to invasive infection. The primary reservoir for commensal *S. aureus* is the vestibulum nasi, which is persistently colonized in 20–30% of the healthy population [1,2,3]. Nasal colonization is associated with an increased risk of infection, and infection is typically caused by the colonizing bacterial isolate [4,5]. In contrast to the nose, the bacterium is absent or detected at a low abundancy in the healthy skin microbiota [6]. Notably, the situation is markedly different in patients with atopic dermatitis (AD). Here, *S. aureus* is frequently isolated from skin samples and associated with dysbiosis and a significantly reduced diversity in the skin microbiota [7,8,9]. The prevalence of *S. aureus* on lesional skin is ~70%, depending on the sampling and detection methods, with bacterial densities associated with disease severity [7,10]. Beside the potential role of *S. aureus*, genetic and environmental factors leading to a defective skin barrier and a dominant type 2 immunity (in its acute phase) contribute to disease pathogenesis [11,12,13,14]. Clinically, AD is characterized by periodic flares of red, itchy, dry skin lesions [15,16] affecting mainly infants (15–30%) and 2–10% of adults [17]. The mechanisms of host and niche adaptation in vivo and the environmental stimuli that convert commensal *S. aureus* into pathogens on the atopic skin are incompletely understood. The expression of virulence factors in *S. aureus* is controlled by interactive regulatory systems that allow specific adaptation to changing environments [18,19]. These include the accessory gene regulator (Agr), which controls virulence gene expression in response to bacterial density through a peptide quorum sensing system. Binding of the autoinducing peptide (AIP) leads to the transcriptional activation of a small regulatory RNA (RNAIII) via a classical two-component system (TCS) [18,20]. RNAIII, in turn, regulates the transcription of various virulence factors by switching the bacterium from a high to a low adhesion state [21], downregulating the expression of cell surface adhesins, and inducing the transcription of toxin genes such as the *hla*-encoding α-toxin (Hla) [22]. The α-toxin is known to damage and lyse keratinocytes, potentially contributing to the pathogenesis of AD [23,24,25]. In addition to RNAIII, the response regulator AgrA (of the TCS) induces the transcription of *psm*-encoding phenol-soluble modulins (PSMs) by directly binding to the promoter region [26]. PSMs are known to induce the release of proinflammatory cytokines in keratinocytes and, thus, could also contribute to the pathogenesis of AD [27,28,29]. It has been shown that the expression of a functional Agr system in *S. aureus* was required for epidermal colonization and the induction of AD-like inflammation in mice [30]. However, the Agr system was found to be primarily inactive during the onset and persistent colonization of the nose [31,32] as well as on human skin explants [33]. Subtle changes in the host environment may result in major changes in gene expression, resulting, for example, in Agr activation during skin colonization in patients with AD. The regulatory processes that facilitate the transition from an asymptomatic nasal colonizer to a pathogen associated with symptomatic skin disease remain unknown. To gain insight into the changes in this process, in this study, we comparatively analyzed bacterial gene expression in nasal and skin swabs from patients with AD by quantitative real-time PCR. The primary challenge in the analysis of gene expression in microbial communities on the skin is the low bacterial burden [34], which has, thus far, limited the application of RNA sequencing technology in patients with AD. To date, only one study has quantified superantigens and toxins at the protein level using a Western dot blot assay [35], while another has employed the preamplification of cDNA prior to qPCR [36]. In addition, both studies lack reference to the primary reservoir—the human nose—which we consider in this study to be the two major habitats where transmission occurs in patients with AD. Specifically, our transcript analysis was conducted without any subcultivation of the bacteria or additional amplification steps of the RNA or cDNA, respectively. This approach serves to minimize the potential biases introduced by these steps, thereby providing a more accurate representation of the transcriptional profile in an authentic human environment. The present study addresses the existing gap in understanding the transcriptional differences between asymptomatic nasal colonization and symptomatic skin colonization/infection within the same individual with AD.

## 2. Results and Discussion

### 2.1. Characteristics of Nasal and Skin Specimens from Patients with AD

**Figure 1:**
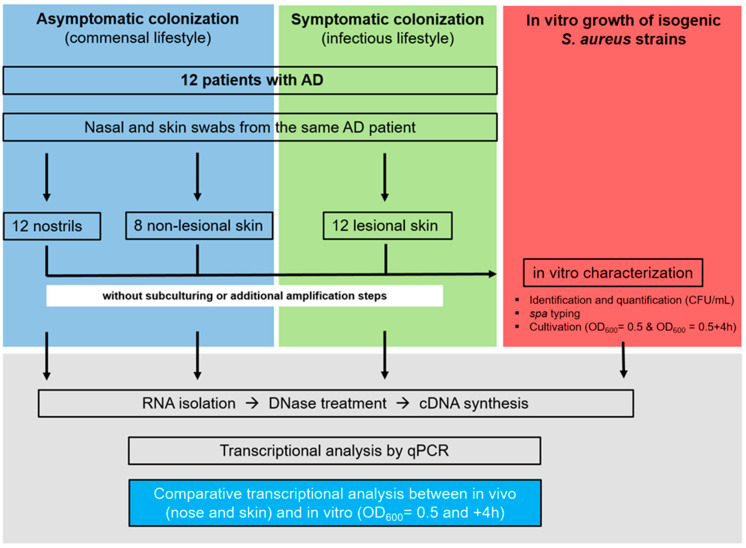
Study design: Nasal, lesional, and non-lesional skin swabs were obtained from 12 patients diagnosed with AD. All the patients were colonized with *S. aureus* in the nose and on the lesional skin. RNA was directly isolated from three consecutive swabs, and bacterial transcription was profiled by qPCR. This allowed for the direct comparison of nasal and skin transcripts within the same patient. In parallel, 10 µL of swab material from each patient was used for in vitro characterization (quantification and *spa* typing) and cultivation to exponential (OD_600_ = 0.5) and post-exponential (OD_600_ = 0.5 + 4 h) growth phases. RNA was isolated, and bacterial transcription was measured by qPCR. The in vivo gene expression in the nose and skin was then compared to the in vitro expression pattern.

To comparatively analyze bacterial gene expression during colonization as a commensal bacterium versus symptomatic skin infection in the same individual, we selected twelve patients with atopic dermatitis (A to L). Swabs from lesional and non-lesional skin sites and from the nostrils were obtained (Figure 1). Since lesional skin is clinically and histologically characterized by inflammation, manifested by immune cell infiltration, acanthotic thickening of the epidermis, changes to the extracellular matrix composition, clinical symptoms such as redness, swelling, and itching [15,37], and the presence of potentially invasive bacteria such as *S. aureus*, we refer to this as infection. In contrast, non-lesional skin does not display any inflammatory responses, and the skin microbiota is less frequently dominated by *S. aureus*. Here, the bacterium colonizes the skin without apparent tissue alterations or even damage caused by the host response. We, therefore, refer to this situation as colonization. 

**Figure 2:**
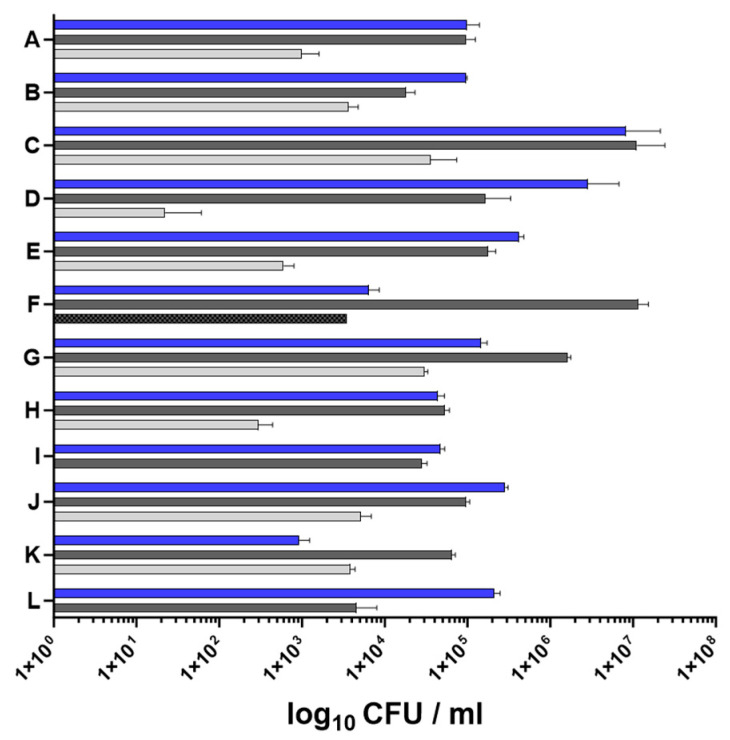
*S. aureus* load in the nose and skin of 12 patients with AD (A–L): The bacterial load in the nose (blue bars), on lesional skin (dark gray bars), and on non-lesional skin (light gray) was determined by the serial dilution and plating of the swab material. Patient F was infected with a hemolytic (dark gray bar) and a non-hemolytic (dark gray bar with black squares) strain of *S. aureus* on lesional skin. CFU, colony-forming units.

All patients were positive for *S. aureus* in the nose and on lesional skin. Nine individuals were also positive for *S. aureus* on non-lesional skin sites. However, in one of these nine patients, the *S. aureus* levels on non-lesional skin were below the detection limit for gene expression analysis (patient D < 100 CFU/mL) (Figure 2).

The *S. aureus* loads in nasal and skin specimens were determined by serial dilution and plating, and they varied widely among the patients, with the lowest bacterial load found on non-lesional skin (mean value of non-lesional skin 8.95 × 10^3^ CFU/mL). No significant difference was observed between the nostrils and the lesional skin sites (mean value of the nose 1.06 × 10^6^ CFU/mL and mean value of lesional skin 1.42 × 10^6^ CFU/mL) (Figure 2).

Phenotypic and genotypic characterization of the *S. aureus* isolates revealed that most patients were colonized with a single *S. aureus* strain. Only patient F simultaneously carried a hemolytic and a non-hemolytic strain on lesional skin. The non-hemolytic isolate differed in the *spa* type (Table 1) and was significantly less abundant than the hemolytic strain (mean abundance of the hemolytic strain 1.17 × 10^7^ CFU/mL versus mean abundance of the non-hemolytic strain 3.53 × 10^3^ CFU/mL). In all the patients except for D and L, the *S. aureus* strain on the skin was identical to the strain in the nose (Table 1). The detection of genes encoded on mobile genetic elements revealed that, with the exception of patient C, all the isolates were lysogenic for *hlb*-converting phages encoding staphylokinase (*sak*), the staphylococcal complement inhibitor (*scn*), and/or the chemotaxis inhibitor protein (*chp*) (Table 1). 

In summary, our data indicate that the *S. aureus* strain present in the nose is frequently identical to that on the skin, consistent with findings of Clausen and colleagues [38]. Since hands are considered to be the primary means of transmitting *S. aureus* from surfaces to the nose [1] and, as it turned out, not only the nose but also the skin serves as a crucial reservoir of *S. aureus* in patients with AD [39], bidirectional transmission between the nose and the skin seems to be most likely.

### 2.2. In Vivo Transcriptional Profiling of S. aureus during Asymptomatic Nasal Colonization to Skin Colonization/Infection in Patients with AD

The aim of this analysis was to obtain insight into the transition from the commensal to the infectious lifestyle by comparatively analyzing samples from both asymptomatic colonization (nose and non-lesional skin) and symptomatic skin infection (lesional skin) in the same patient with AD. For the direct in vivo transcript analysis, three consecutive swabs collected from the three anatomical sites were analyzed without any cultivation or amplification step. Bacterial mRNA expression was quantified in the in vivo specimen and compared to the transcript expression during the in vitro cultivation of the same *S. aureus* isolate (Figure 1). For *S. aureus* isolates with a different *spa* genotype from the nose and the skin (patients D and L), transcription was compared to the isogenic isolate (Table 1). 

#### 2.2.1. *S. aureus* Agr Activity Is Increased in the Skin Samples of Some Patients with AD

**Figure 3:**
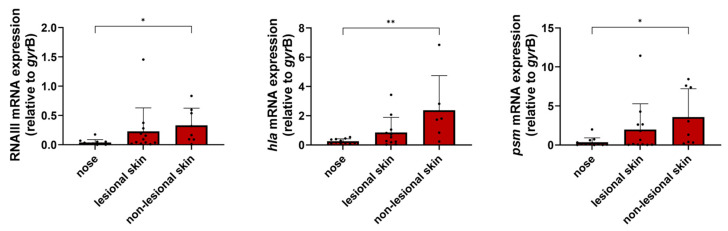
Transcriptional analysis of Agr target genes (RNAIII, *hla*, and *psm*) in swab material of the nose and skin of 12 patients with AD. The transcripts were quantified relative to the transcription of the housekeeping gene *gyr*B directly in the nose and skin swabs. The dots represent individual patients. Statistically significant differences are indicated as follows: * *p* ≤ 0.05 and ** *p* ≤ 0.01.

Regulatory elements, such as the accessory gene regulator Agr, play a critical role in the virulence regulation of *S. aureus* [18,40]. Here, we analyzed the transcriptional activity of the Agr quorum sensing system by quantifying the transcription of three validated target genes, including the effector molecule RNAIII, *hla,* and *psm*. We were able to verify previous results from healthy individuals indicating that none of the analyzed Agr target genes are expressed during the colonization of the nose [31,32]. However, the transcriptional activity of the Agr target genes on the skin of patients with AD was strongly increased in three patients (patients B, E, and I) and moderately elevated in three others (patients D, G, and H) (Supplementary Figure S1). Interestingly, transcription was even stronger in the non-lesional skin samples and did not correlate with the *S. aureus* load (Figure 2). The overall statistical analysis of the data from all 12 patients with AD revealed a consistent trend towards greater Agr activity on the skin compared to the nose in the in vivo situation. This trend reached statistical significance for all three target genes on non-lesional skin (Figure 3). This significant increase in the transcriptional activity of Agr was unexpected and currently lacks an explanation. The microbial communities of non-lesional skin sites are less dominated by *S. aureus* and exhibit greater diversity than those of lesional skin sites [41]. This difference in microbiota composition may result in increased Agr activity by microbiota signals on non-lesional skin and, in turn, is necessary for the persistence of *S. aureus* in this habitat. This indicates that properties of the skin environment per se trigger Agr activity independent of inflammation or bacterial density. Since the skin of healthy individuals is rarely colonized by *S. aureus* [6,42,43], it remains cryptic whether this finding is specific for patients with AD. However, initial contact with healthy human skin resulted in a significant downregulation of Agr in our recently developed ex vivo colonization model [44]. This discrepancy in Agr activity may be due to differences in the anatomical or physiological characteristics of healthy versus atopic skin or the kinetics of colonization. Since the clinical samples were collected irrespective of disease severity and at different disease stages (acute phase, after a flare, or with mild or almost no symptoms) as well as independent of the therapeutic regimen, the heterogeneity of *agr* expression might be attributed to the diverse stages of infection of both non-lesional and symptomatic atopic skin as well as the variable disease severity. Cruz and colleagues have shown that the transcriptional activity of Agr is downregulated upon initial contact with disrupted human skin (barrier dysfunction induced by tape stripping) but increases 24 h after colonization [45]. Thus, Agr activity may be dynamic during skin colonization/infection. It remains unclear why skin-specific Agr activation was observed only in a subgroup of patients with AD. This may be attributed to the *S. aureus* strains, with potentially only certain strains responding to the “skin signal”. Therefore, future studies, for example, directly after successful therapy, will need to identify when Agr and its target gene expression are beneficial by collecting follow-up isolates, as well as which external and environmental stimuli cause activation, particularly in a non-lesional skin environment. 

Evidence exists that the dynamic activation of Agr is critical for the formation of abscesses in mice [46], and Agr may also contribute to skin colonization in patients with AD, which is a highly dynamic disease with relapsing flares [47]. Consistently, a study by Nakamura and colleagues demonstrated that the Agr system drives *S. aureus* skin colonization in infants with AD [30]. In a Japanese cohort, children colonized with *S. aureus* at 6 months of age had a greater risk of developing AD. Furthermore, whereas mutations in the Agr system were detected in *S. aureus* strains colonizing healthy skin, they were not detected in children who developed AD [30]. The same group also demonstrated that the Agr system plays a critical role in epidermal colonization and the development of AD-like inflammation in a murine epicutaneous colonization model [30,48]. In contrast, Soong and colleagues isolated *S. aureus* strains that contained an *agr* mutant-like phenotype in 22% of chronically infected patients with AD [49]. According to the authors, this adaptation to human skin and the occasional selection of *agr* mutants following persistent infection through intracellular localization in keratinocytes may promote chronic infection in AD [49]. By analyzing the initially contradictory results of the two groups, substantial evidence has emerged to support the notion that Agr plays a critical role during the development of AD per se and that the presence of Agr mutants favors a chronic course of infection. 

In summary, differences in Agr activity mediated both through point mutations as well as on the transcriptional level, seem to be important to facilitate infection of human skin in patients with AD. Our results suggest that Agr activity and the resulting toxin expression may play a critical role in the transition from asymptomatic nasal colonization to skin colonization/infection in patients with AD. 

#### 2.2.2. Gene Expression in the Nasal Cavity and Skin Environment Appears Similar

For further comparison, we examined the expression of 29 genes from nose and skin isolates. We selected genes coding for virulence regulators, toxins, adhesins, and factors involved in cell wall dynamics, immune evasion, protease activity, and metabolism. The results were visualized in a heat map showing the in vivo transcription (in the nasal, lesional, and non-lesional skin specimens) relative to the maximum expression of the isogenic *S. aureus* strain grown in vitro (OD_600_ = 0.5 or OD_600_ = 0.5 + 4 h). In addition, to test general differences and trends for statistical significance, we summarized the expression data from all the patients and compared the transcription between the different habitats and in vitro. In the following, the results are discussed according to functional categories. 

##### Global Transcriptional Regulators

**Figure 4:**
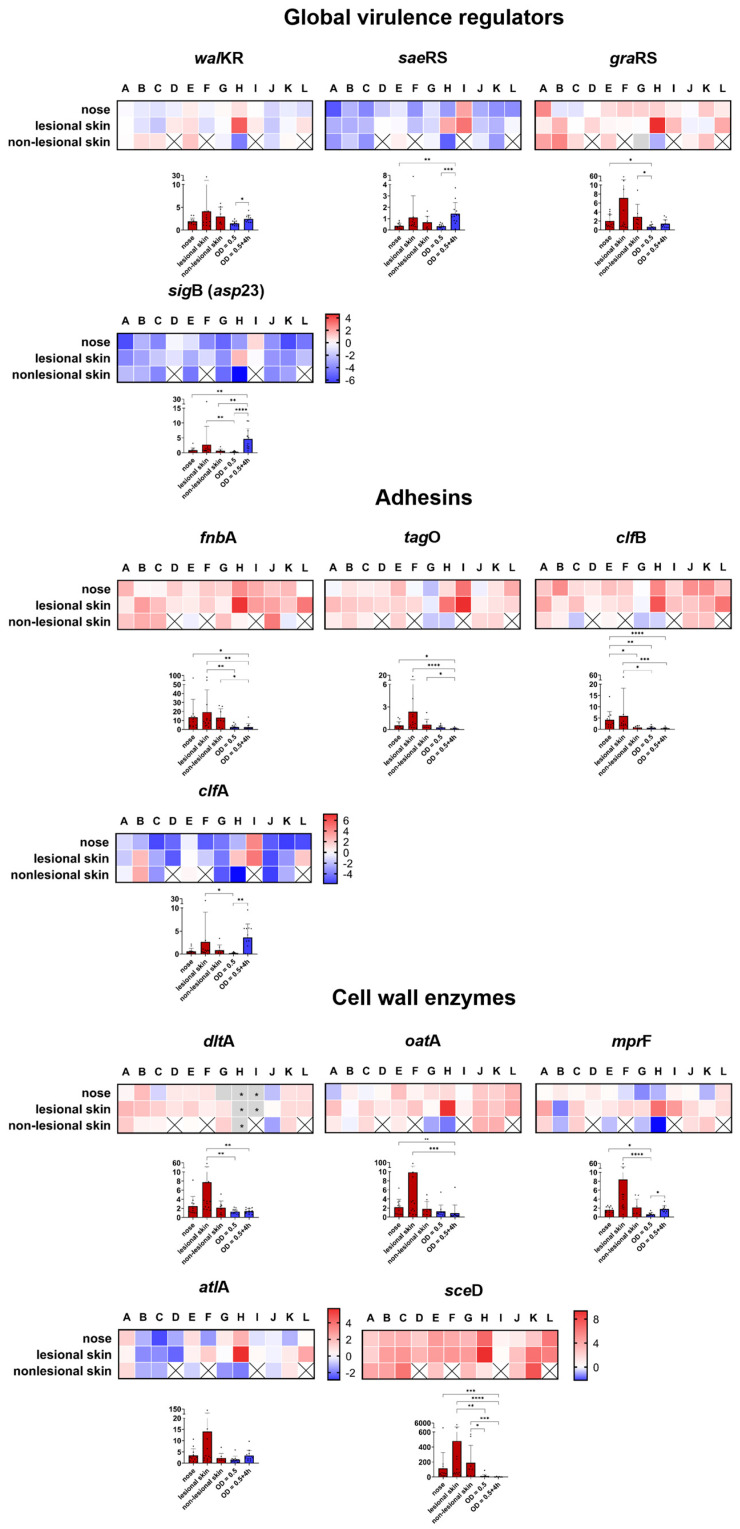
Direct transcript analysis of *S. aureus* genes in the nose and skin of 12 patients with AD (A–L). The results presented as a heat map are given as the ratio of transcription in vivo versus the maximal expression in vitro. All the data were log transformed (basis 2), and changes in gene expression were normalized in reference to the constitutively expressed gene *gyr*B. The genes in red are those which were upregulated compared to the in vitro conditions, and the genes in blue are those which were downregulated compared to the in vitro conditions. The white cells indicate the same expression levels in vivo and in vitro. The gray cells indicate that in vivo transcription was below the threshold of detection. The gray cells with an asterisk indicate that either the in vitro or in vivo values were excluded, and, therefore, no ratio could be calculated. The results are the means of three separate samplings. The white cells with an “x” indicate that the gene is not present in the strain or that no *S. aureus* was detected in the corresponding habitat (non-lesional skin). The column diagrams indicate the combined transcription of all 12 patients with AD directly in the nose and skin swabs (red columns) and in vitro (blue columns). Each dot represents an individual patient. * *p* ≤ 0.05; ** *p* ≤ 0.01; *** *p* ≤ 0.001; and **** *p* ≤ 0.0001.

Expression of the virulence regulator *sae*RS (*S. aureus* exoprotein expression) and the alternative sigma factor B (*sig*B) (detected as the tightly *sig*B-dependent gene *asp*23) was low in all three in vivo specimens. Only patients D, H, and I showed elevated *sae* and *sig*B transcription in the nose and/or lesional skin (Figure 4). The overall low level of *sae* transcriptional activity is consistent with the reduced transcription observed in our ex vivo colonization model [44]. Considering the low transcriptional levels found in the nose of patients with AD and healthy individuals [32], it appears that both regulatory elements have a minimal impact during colonization and atopic skin infection. In contrast, the transcriptional activity of the two- and three-component systems, *wal*KR and *gra*XRS, respectively, was increased primarily on the skin, with *gra*XRS showing less heterogeneity between patients (Figure 4). Of note, the *wal*KR and *gra*XRS target genes *sce*D (Staphylococcus carnosus exoprotein D), *dlt*A (D-alanine-D-alanyl carrier protein ligase), and *mpr*F (multiple peptide resistance factor) also showed increased expression (Figure 4). The statistical analyses indicated an expected general trend in terms of a significantly higher in vitro expression of all global virulence regulators during the post-exponential growth phase. However, no significant differences were observed between the habitats of the nose and the skin (Figure 4 and Supplementary Figure S2).

##### Adhesins

The attachment of *S. aureus* to host structures during colonization and infection is mediated by a plethora of adhesion molecules [50,51,52]. These include classical adhesins, which are cell wall-anchored (CWA) proteins [50], but also structures such as wall teichoic acid (WTA), which mediate adhesion to epithelial and endothelial cells [53]. The mechanisms underlying massive *S. aureus* infection of the skin of patients with AD, especially during a flare, are not fully understood. In addition to the low colonization resistance mediated by the significantly altered skin microbiota [8,54], factors such as changes in the stratum corneum and the morphology of corneocytes [37] may explain the observed preferential adherence and colonization. It has been shown that *S. aureus* fibronectin-binding proteins (FnBPs) mediate the stronger adhesion to AD skin biopsies, which may be due to the presence of fibronectin (the ligand of *S. aureus* FnBPs) in the stratum corneum of AD skin but not healthy skin [55]. In fact, our transcriptional analysis of fibronectin-binding protein A (*fnb*A), in addition to the nose, revealed increased expression in both the lesional and non-lesional skin specimens. This increase was found to be statistically significant in comparison to the maximal expression observed during growth in vitro (Figure 4). Additionally, significantly increased transcript levels (in comparison to in vitro) of other adhesion-mediating molecules, such as clumping factor B (*clf*B) and WTA-synthesizing enzymes (measured as *tag*O), were observed. Transcription on non-lesional skin exhibited a slight variation among patients (Figure 4). All these molecules are known to play a critical role in nasal colonization [32,56,57]. The high transcription levels in the nose of patients with AD (Figure 4) in this study confirm this finding. Compared to all other analyzed adhesion molecules, which were not significantly differentially transcribed between the habitats (Supplementary Figure S2), only *clf*B exhibited a significant downregulation on non-lesional skin compared to the nose and lesional skin (Supplementary Figure S3). 

Clumping factor A (*clf*A), an adhesion expressed in vitro during the late growth phase [58], was weakly transcribed in the majority of patients, with the exception of patients B, E, H, I, and L, who exhibited higher levels of transcription primarily on lesional skin (Figure 4). Increased transcription of *clf*A was observed in our ex vivo human skin model [44] and in the barrier-disrupted skin model of Cruz et al. [45], suggesting involvement during the early phase of colonization. A temporal expression of the adhesion factor WTA towards *clf*B has already been detected during the establishment of colonization in the cotton rat nose [31]. 

In summary, similar adhesive molecules seem to participate in nasal colonization and skin infection, as indicated by comparable expression patterns in both niches.

##### Cell Wall Modification Enzymes

Enzymes involved in remodeling the bacterial cell wall are critical for colonization [59,60] as they mediate, for example, resistance to antimicrobial peptides (AMPs). Both the *dlt* operon, which mediates the D-alanylation of teichoic acids, and the *mpr*F-mediated incorporation of lysyl-phosphatidylglycerol into the cytoplasmic membrane mediate resistance to cationic AMPs by generating a positively charged surface envelope [61]. Here, we detected increased levels of *dlt*A and *mpr*F transcription on lesional skin. While *dlt*A was also highly expressed in the nose and on non-lesional skin, *mpr*F revealed greater heterogeneity at these anatomical sites (Figure 4). Notwithstanding the aforementioned greater heterogeneity, the *mpr*F gene is the only gene within this analyzed category that exhibits a statistically significant difference in transcription between the nose and lesional skin (Supplementary Figure S3).

Additionally, an elevated transcription of O-acetyltransferase A (*oat*A), which mediates resistance to the lysozyme [62], was detected, whereas expression of the major autolysin *atl*A showed a high degree of variation between patients with AD (Figure 4). The most pronounced transcriptional increase was observed for the lytic transglycosylase *sce*D (Figure 4). A significant increase in *sce*D was also observed during the initial stages of skin colonization [44]. The expression level of *sce*D is typically low during growth in rich media in vitro, but it was high in both lesional and non-lesional skin. This suggests that our ex vivo tissue model, as recently reported, mimics the situation in humans. Additionally, it suggests that *sce*D plays a critical role in the infection process of *S. aureus* in patients with AD. The marked increase in *sce*D expression, observed in multiple anatomical sites, model systems, and across various species, including *S. epidermidis* (reviewed in [33]), highlights the potential of this protein as a target for antistaphylococcal therapy not only in patients with AD. Furthermore, it underscores the significance of cell wall dynamics during host–pathogen interactions.

##### Immune Evasion

**Figure 5:**
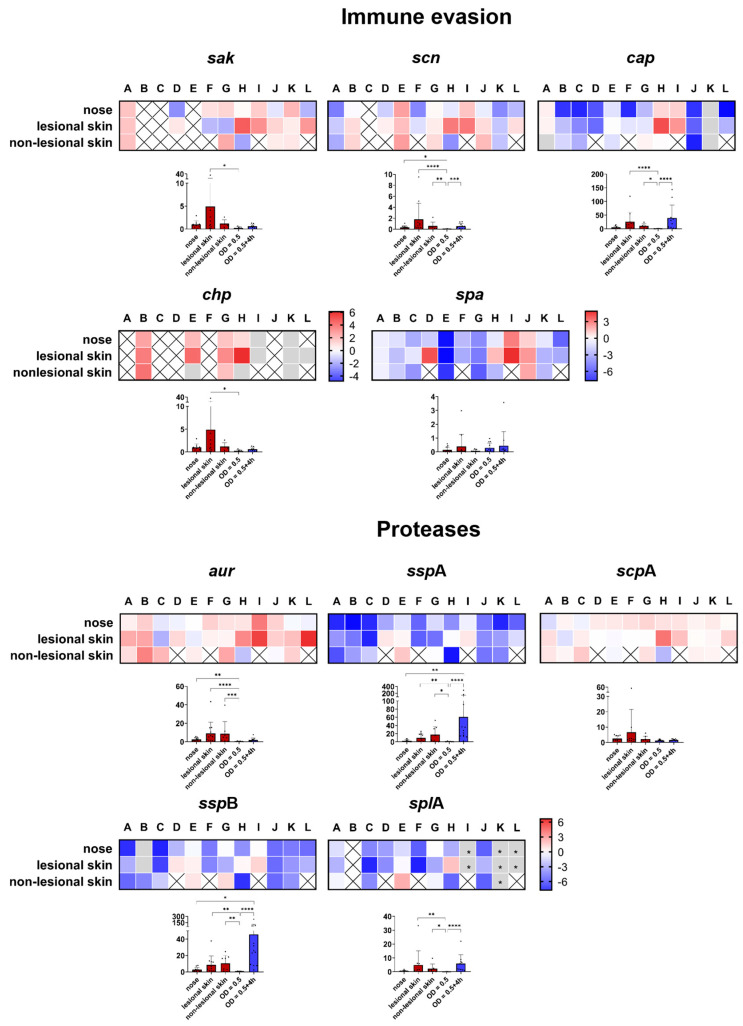
Direct transcript analysis of *S. aureus* genes in the nose and skin of 12 patients with AD (A–L). The results presented as a heat map are given as the ratio of transcription in vivo versus the maximal expression in vitro. All the data were log transformed (basis 2), and changes in gene expression were normalized in reference to the constitutively expressed gene *gyr*B. The gray cells indicate that the in vivo transcription was below the threshold of detection. The gray cells with an asterisk indicate that either the in vitro or in vivo values were excluded, and, therefore, no ratio could be calculated. The results are the means of three separate samplings. The white cells with an “x” indicate that the gene was not present in the strain or no *S. aureus* was detected in the corresponding habitat (non-lesional skin). The column diagrams indicate the combined transcription of all 12 patients with AD directly in the nose and skin swabs (red columns) and in vitro (blue columns). Each dot represents an individual patient. * *p* ≤ 0.05; ** *p* ≤ 0.01; *** *p* ≤ 0.001; and **** *p* ≤ 0.0001.

Evasion of the human immune system by *S. aureus* involves numerous secreted and cell wall-associated virulence factors [63,64,65]. Among these factors, protein A (*spa*) and the extracellular capsular polysaccharide (*cap*) revealed low transcriptional levels during both asymptomatic colonization and infection in patients with AD. Nevertheless, *spa* and *cap* showed greater interindividual variation between patients; for example, patients H and I revealed increased transcription levels compared to the maximal expression of their isogenic isolates (Figure 5). The low transcription level of both molecules is consistent with low expression during the initial colonization of healthy human skin [44]. In contrast, the transcription of the phage-encoded immune evasion molecules, including staphylokinase (*sak*), the chemotaxis inhibitory protein (*chp*), and the staphylococcal complement inhibitor (*scn*), exhibited increased transcript levels, with *chp* being most consistently elevated in all habitats, irrespective of skin barrier integrity (Figure 5).

Thus, despite some heterogeneity between the patients, all three phage-encoded genes seem to participate in inhibiting the complement cascade during skin infection in patients with AD. In contrast, it appears that the encapsulation and binding of antibodies by protein A and, therefore, the inhibiting of complement activation via C1q as well as phagocytosis [66,67] are not primarily involved in the development of AD. With the exception of *cap*, which shows a significant upregulation on lesional skin compared to the nose (Supplementary Figure S3), no significant differences between the three habitats were observed for the other genes (Supplementary Figure S2).

##### Proteases

Proteases produced by *S. aureus* are believed to significantly contribute to the development of AD. This may occur by either mediating immune evasion [68,69] or by disrupting the skin’s epidermal barrier [70], subsequently enabling bacterial penetration into deeper layers of the epidermis [71]. However, it remains unknown which *S. aureus* proteases are specifically implicated in the pathogenesis of AD. Therefore, we analyzed genes encoding all three catalytic classes of proteases. We observed a distinct transcriptional pattern in the skin of patients with AD (Figure 5). While the metalloprotease aureolysin (*aur*) and the cysteine protease staphopain A (*scp*A) were highly expressed in the skin specimens, the cysteine protease staphopain B (encoded by *ssp*B) and the serine protease *ssp*A (V8 protease) and *spl*A (member of the serine protease-like proteins) were only weakly transcribed. Compared to its in vitro expression, the transcription of aureolysin reached statistical significance in all three habitats (Figure 5). Aureolysin promotes the survival of *S. aureus* by degradation of the antimicrobial peptide LL-37 [68] found on the skin [72] and in the human nasal mucosa [73]. Furthermore, the metalloprotease cleaves the central complement protein C3, which affects complement activation [74] and, therefore, may act in concert with the aforementioned phage-encoded molecules SAK, SCN, and CHIP to achieve immune evasion. Staphopain A is known to cleave elastins [75] and inactivate several host proteins, thereby directly affecting the host’s connective tissue and coagulation systems [76,77].

In summary, our study provides evidence for the direct involvement of some proteases most likely to promote *S. aureus* survival on the skin of patients with AD. Due to the diverse functions of proteases in regulating both the *S. aureus* proteome and the host’s defense molecules, selective expression appears to be crucial in vivo. Between the different habitats, no significant differences in the transcript levels were observed (Supplementary Figure S2).

##### Genes Involved in Iron Limitation and Cell Respiration

**Figure 6:**
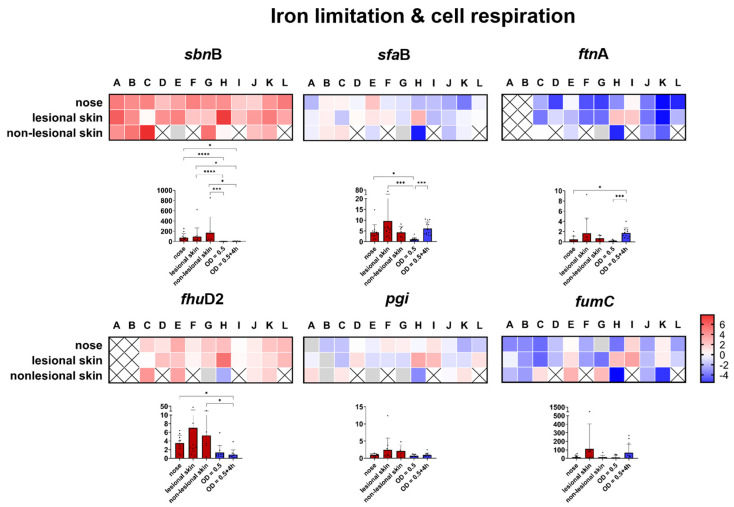
Direct transcript analysis of *S. aureus* genes in the nose and skin of 12 patients with AD (A–L). The results presented as a heat map are given as the ratio of transcription in vivo versus the maximal expression in vitro. All the data were log transformed (basis 2), and changes in gene expression were normalized in reference to the constitutively expressed gene *gyr*B. The gray cells indicate that in vivo transcription was below the threshold of detection. The results are the means of three separate samplings. The white cells with an “x” indicate that the gene was not present in the strain or no *S. aureus* was detected in the corresponding habitat (non-lesional skin). In patients A and B, the quantity of sample material available for analysis of the *ftn*A and *fhu*D2 genes was insufficient. The column diagrams indicate the combined transcription of all 12 patients with AD directly in the nose and skin swabs (red columns) and in vitro (blue columns). * *p* ≤ 0.05; *** *p* ≤ 0.001; and **** *p* ≤ 0.0001.

Similar to the selective expression pattern of the proteases, expression of the siderophores staphyloferrin A and B also displayed a high selectivity. While staphyloferrin B (measured by *sbn*B) was significantly expressed in all patients, staphyloferrin A (measured by *sfa*B) exhibited a high variability in expression (Figure 6). Furthermore, no significant differences in the transcript levels were observed between the different habitats (Supplementary Figure S2). The ferric hydroxamate siderophore-binding protein (*fhu*D2), which mediates iron uptake by binding to xeno hydroxamates produced by other microorganisms [78], was highly expressed in all patients (Figure 6). Low expression of the storage ferritin protein *fnt*A serves as another indicator of iron limitation, with its expression being repressed under conditions of iron deficiency [79]. In addition to clear iron limitation, the activity of the glycolysis and TCA cycles did not seem to follow a trend (Figure 6).

## 3. Materials and Methods

### 3.1. Ethics Statement

The skin and nasal swabs were obtained from patients with atopic dermatitis. This approach was approved by the local ethics committee of the Medical Faculty RWTH University of Aachen, Germany (EK 217/19). In accordance with the Declaration of Helsinki Principles guidelines, written consent was obtained from all the participants involved in this study.

### 3.2. Study Population

Twelve patients with AD (four females and eight males, median age 41.5 years [range 22–65 years]) were included in this study. The patients were recruited by the treating physician during their routine clinic visit and were included regardless of the therapeutic regimen. The overall disease severity of the patients with AD was assessed using the SCORing Atopic Dermatitis (SCORAD). All patient characteristics are shown in Table 1.

### 3.3. Sampling, Bacteriological Analysis, and Growth of S. aureus Strains

For the in vivo transcript analysis, a cotton wool swab was moistened with 300 µL of nuclease-free water (Thermo Fischer Scientific, Waltham, MA, USA), and the left and right anterior nares of the patients were sampled. Skin swabs were obtained by rotating the swab on lesional and non-lesional skin sites. The swab was vigorously vortexed, and 10 µL of the suspension was used for quantitative bacteriological analysis. The samples were analyzed on sheep blood agar plates (Thermo Fischer Scientific, Waltham, MA, USA) by serial dilution and the plating of the swab material, and *S. aureus* was identified by Staphaurex (Thermo Fischer Scientific, Waltham, MA, USA). The cotton wool was removed from the swab using sterile tweezers, and both the suspension and the cotton wool were directly stored in 1 mL of TRIzol LS reagent ((Thermo Fischer Scientific, Waltham, MA, USA)) containing 0.5 mL of zirconia/silica beads (0.1 mm in diameter [Carl Roth GmbH + Co. KG, Karlsruhe, Germany]).

For the in vitro transcript analysis, the clinical *S. aureus* isolates were grown overnight in TSB medium (Carl Roth GmbH + Co. KG, Karlsruhe, Germany), diluted to an initial optical density value at 600 nm (OD_600_) of 0.05 in fresh medium and grown with shaking (220 rpm) at 37 °C until the exponential (OD_600_ = 0.5) and post-exponential (OD_600_ = 0.5 + 4 h) phases. Bacteria were harvested by centrifugation and dissolved in 1 mL TRIzol reagent containing 0.5 mL of zirconia/silica beads.

### 3.4. RNA Isolation, Reverse Transcription, and Quantitative Real-Time PCR

Bacterial lysis, RNA isolation, DNase treatment, and reverse transcription were performed as described previously [32,80]. The relative quantification of the *S. aureus* transcripts was carried out using the QuantStudio 1 Real-Time-PCR-System. Master mixes were prepared as follows: 8 µL KAPA SYBR^®^ FAST qPCR Master Mix (2×) ABI Prism (Roche, Basel, Switzerland), 8 µL nuclease-free water, 1 µL of specific primer published in [31,32,44,81] or listed in Supplementary Table S1. The following temperature profile was utilized for amplification: denaturation for 1 cycle at 95 °C for 15 s, 55 cycles at 95 °C for 27 s, 55–60 °C for 10 s, and 72 °C for 27 s, with fluorescence acquisition at 72 °C. The melting curve analysis was carried out at 60–97 °C with stepwise fluorescence acquisition. Finally, 2 µL of cDNA was added per reaction. The relative quantities of the transcripts were calculated by a standard curve for each gene generated using a 6-fold serial dilution of an *S. aureus* USA300 wild-type cDNA mixture from the exponential and post-exponential phases.

In addition to the melting curve analysis, the specificity of the quantitative real-time PCR reaction was verified for some genes using 3% agarose gels. These gels included, in addition to the in vivo samples and the corresponding *S. aureus* isolates grown in vitro from samples obtained from patients with AD, the *S. aureus* USA300 relative standard transcripts as a positive control and a negative control of the qPCR master mix without cDNA. All the primers were designed for optimal binding to the genome-sequenced *S. aureus* strains in silico, and possible cross-reactions with other bacterial species, such as *S. epidermidis*, were excluded. The absence of contaminating DNA was confirmed through quantitative real-time PCR using *gyr*B-specific primers. No amplification products were observed in any of the samples tested.

### 3.5. Spa Genotyping 

For PCR amplification of the *S. aureus* protein A (*spa*) repeat region, a *S. aureus* colony was added to a 0.2 mL PCR tube and heated for 3 min in the microwave at the highest power setting. A 25 µL master mix containing 17 µL nuclease-free water, 2.5 µL 10× PCR Buffer (Qiagen, Hilden, Germany), 2 µL dNTP mix (10 mM each) (Thermo Fischer Scientific, Waltham, MA, USA), 1 µL MgCl_2_ (25 mM) (Qiagen, Hilden, Germany), 1 µL of each primer published in [82], and 0.5 µL HotStarTaq DNA Polymerase (5 units/µL) (Qiagen, Hilden, Germany) was then added. The thermal cycling parameters included the following: an initial 15 min. at 95 °C; 35 cycles of 30 s at 95 °C, 30 s at 55 °C, and 45 s at 72 °C; and a final extension at 72 °C for 10 min. The PCR products were purified with the innuPREP PCRpure Kit (Analytik Jena, Jena, Germany), according to the manufacturer’s instructions, and sequenced by a commercial supplier (4base lab AG advanced molecular analysis, Reutlingen, Germany). The sequences were analyzed with the Ridom StaphType software [https://www.ridom.de/staphtype/ (accessed on 18 August 2024)] (Ridom GmbH, Münster, Germany). The MLST clonal complexes (CCs) were deduced from the BURP grouping of spa types using the Ridom SpaServer database (http://www.spaserver.ridom.de (accessed on 18 August 2024)).

### 3.6. Data Visualization and Statistical Analysis

Differential gene expression as the ratio between the in vivo values and the maximum expression in vitro was visualized in heat maps generated using GraphPad Prism 9.0.2. The calculations were performed as described in [80]. The genes in red are those which were upregulated compared to the in vitro values, and the genes in blue are those which were downregulated compared to the in vitro values. The white cells indicate the same expression levels in vivo and in vitro. The gray cells indicate that transcription was below the threshold of detection. Statistical analysis was performed with the GraphPad Prism software package (version 9.0.2) using the Kruskal–Wallis test. A value of *p* < 0.05 was considered statistically significant.

## 4. Conclusions

In summary, we hereby provide the first in vivo *S. aureus* gene expression analysis comparing asymptomatic nasal colonization and skin colonization/infection within the same individual. Our results suggest that the Agr peptide quorum sensing system may be involved in the bacterium’s transition from the nose to skin. Surprisingly, it seems that transcription of the Agr target genes was even stronger in the non-lesional skin samples compared to the lesional skin, which may be important for survival within this anatomical niche, where the bacterium is generally absent in healthy individuals.

Unexpectedly, despite significant physiological differences between the analyzed habitats (reviewed in [33]), besides Agr activity, gene expression in the nasal and skin environment appeared similar. Consequently, to gain a more comprehensive understanding of gene expression and identify differences between nose and skin environments, RNA-seq analyses are necessary. Finally, while general trend analysis is important, it is also essential to compare expression levels with their isogenic *S. aureus* strains.

## Figures and Tables

**Table 1:** Patient characteristics and phenotypic and genotypic characteristics of *S. aureus* isolates.

Patient	Age	Sex	SCORAD	Comorbidities	*S. aureus* Isolate	Spa Typing ID	Deduced MLST CC	Accessory Gene Content	Phenotype
A	19	female	77	-	nose lesional skin non-lesional skin	t304	-	*sak*, *scn*	hemolytic hemolytic hemolytic
B	61	female	72.6	Asthma bronchiale Milk product allergy	nose lesional skin non-lesional skin	t1451	-	*chp*, *scn*	hemolytic hemolytic hemolytic
C	39	male	80.4	Asthma bronchiale Rhinoconjunctivitis	nose lesional skin non-lesional skin	t008	CC8	-	hemolytic hemolytic hemolytic
D	36	male	34.4	Asthma bronchiale Rhinoconjunctivitis	nose lesional skin	t674 t1943	-	*sak*, *scn*	hemolytic hemolytic
E	54	male	10.5	Rhinoconjunctivitis	nose lesional skin non-lesional skin	t094	-	*chp*, *scn*	hemolytic hemolytic hemolytic
F	38	female	47	-	nose lesional skin ^1^ lesional skin ^2^	t091 t091 t3697	-	*sak*, *scn*	hemolytic hemolytic non-hemolytic
G	48	male	48	Asthma bronchiale Rhinoconjunctivitis	nose lesional skin non-lesional skin	t190	CC8	*sak*, *chp*, *scn*	hemolytic hemolytic hemolytic
H	41	male	41.3	Asthma bronchiale Contact allergy (Amerchol)	nose lesional skin non-lesional skin	t002	CC5	*sak*, *chp*, *scn*	hemolytic hemolytic hemolytic
I	53	male	48.6	Contact allergy (Isoeugenol)	nose lesional skin	t026	CC45	*sak*, *chp*, *scn*	hemolytic hemolytic
J	19	male	45.5	Asthma bronchiale Rhinoconjunctivitis	nose lesional skin non-lesional skin	t091	-	*sak*, *scn*	hemolytic hemolytic hemolytic
K	38	male	38.5	Asthma bronchiale Rhinoconjunctivitis Contact allergy	nose lesional skin non-lesional skin	t2953	-	*sak*, *chp*, *scn*	hemolytic hemolytic hemolytic
L	35	female	29	Asthma bronchiale Rhinoconjunctivitis	nose lesional skin	t091 t1268	-	*sak*, *scn* *sak*, *chp*, *scn*	hemolytic hemolytic

^1^ hemolytic strain of *S. aureus*; ^2^ non-hemolytic strain of *S. aureus*.

## Data Availability

This study includes no data deposited in external repositories. No large datasets were generated or analyzed.

## Supplementary Materials

The following supporting information can be downloaded at https://www.mdpi.com/article/10.3390/ijms25179165/s1.
