# Supplementary material for: Transcriptional Profiling of Staphylococcus aureus during the Transition from Asymptomatic Nasal Colonization to Skin Colonization/Infection in Patients with Atopic Dermatitis
Source: Int J Mol Sci. 2024 Aug 23;25(17):9165. doi: 10.3390/ijms25179165 (PMC11394835; doi:10.3390/ijms25179165)
Supplement: Supplementary file 1 [file ijms-25-09165-s001.zip › ijms-3148021-supplementary.pdf]

International Journal of
Molecular Sciences

MDPI

Supplementary Materials:

Figure S1:

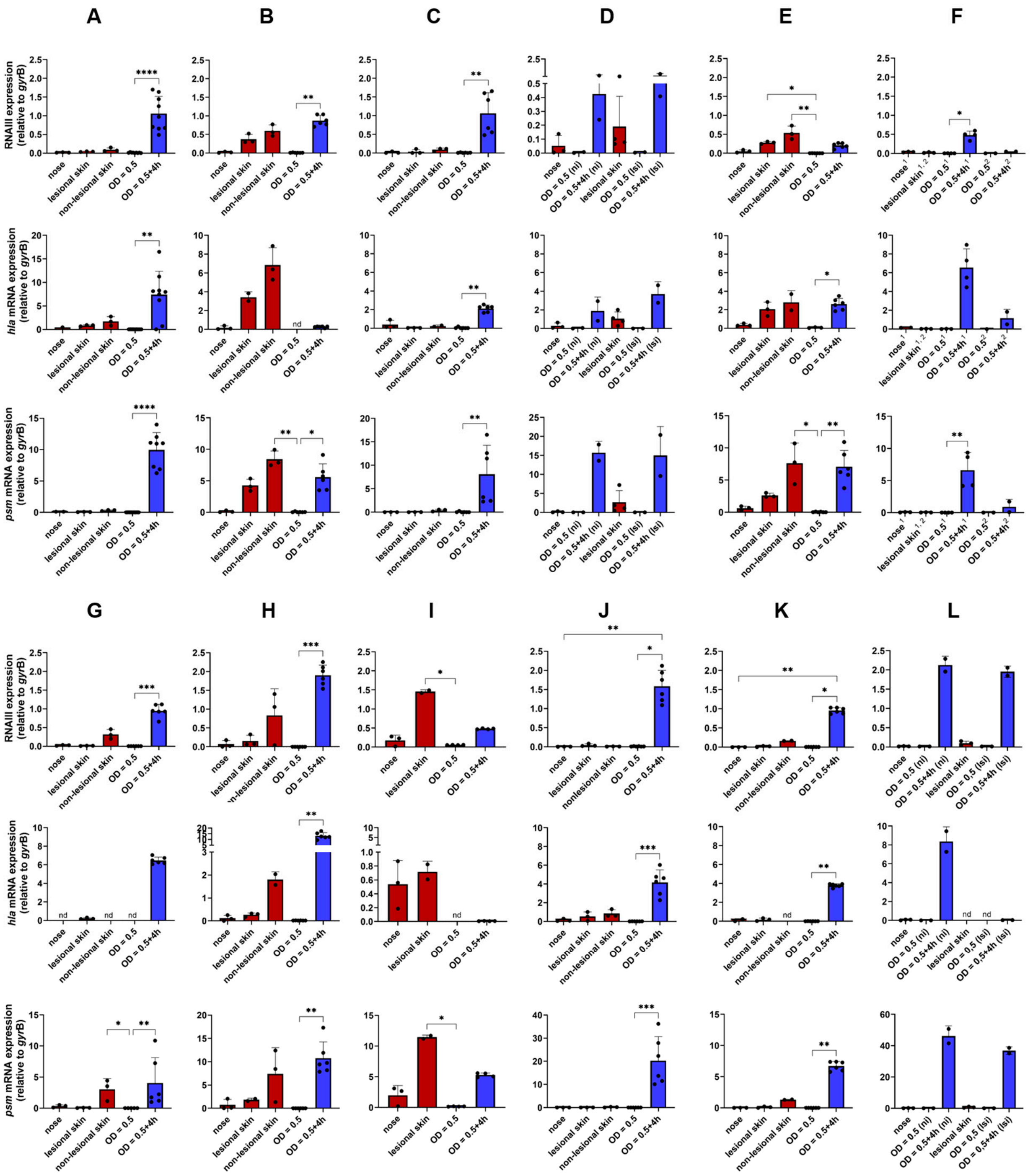

Supplementary Figure S1: Transcriptional analysis of Agr target genes (RNAIII, *hla* and *psm*) in swab material of the nose and skin of 12 patients with AD (A–L). Transcripts were quantified relative to the transcription of the house keeping gene *gyr*B directly in nose and skin swabs (red columns) and after growth in culture (blue columns) to the exponential phase (OD_{600} = 0.5) and the post-exponential phase (OD_{600} =0.5+4h). The hemolytic and non-hemolytic *S. aureus* strains (patient F) are indicated by superscript "1" (hemolytic) and supercript "2" (non-hemolytic). In patients D and F, where the *S. aureus* strains have two different *spa*-types, the nasal strain was labeled "ni = nose isolate" and the lesional skin strain was labeled "lsi = lesional skin isolate". Statistically significant differences between the *in vivo* (nose versus skin) and *in vitro* (*in vivo* versus *in vitro*) results are indicated: P ≤ 0.05; * P ≤ 0.01; ** P ≤ 0.001; *** P ≤ 0.0001; ****, nd, not detectable.

Figure S2:

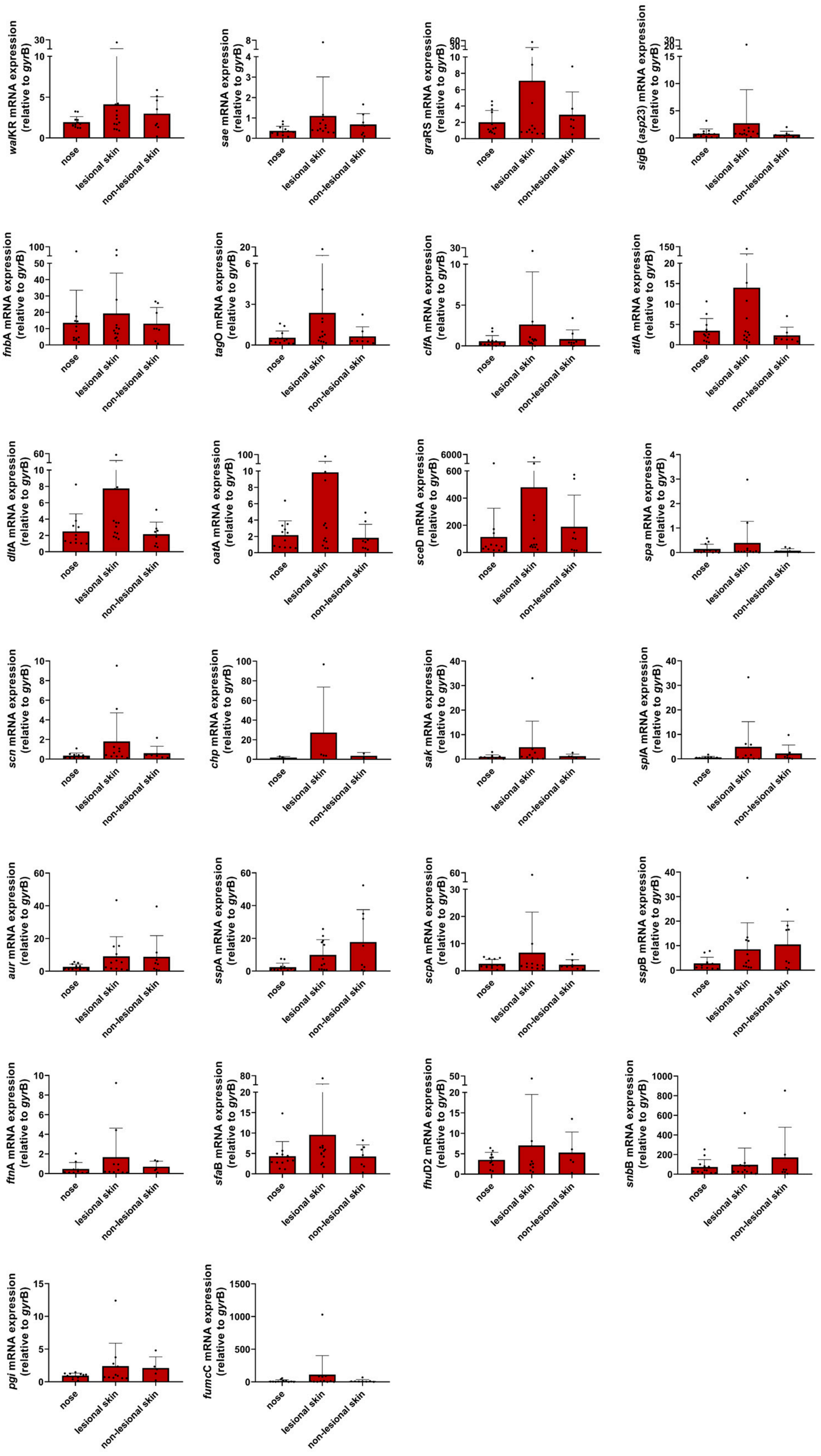

Supplementary Figure S2: *S. aureus* genes with no statistical difference between the habitats. Transcripts were quantified relative to the transcription of the house keeping gene *gyr*B directly in nose and skin swabs of 12 AD patients . The dots represent individual patients.

Figure S3:

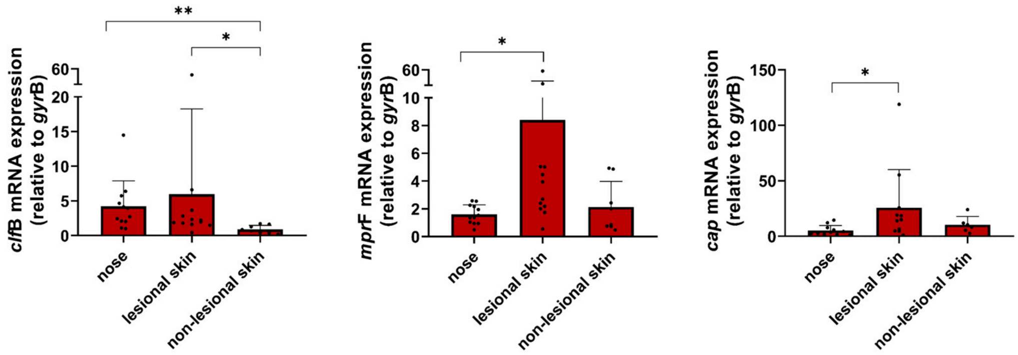

Supplementary Figure S3: ***S. aureus*** **genes with statistical difference between the habitats.** Transcripts were quantified relative to the transcription of the house keeping gene *gyr*B directly in nose and skin swabs of 12 AD patients. The dots represent individual patients. Statistically significant differences are indicated: $P \leq 0.05$; * $P \leq 0.01$; **.

Supplementary Table S1: Oligonucleotide primers

Target gene	Primer	Primer sequence	Purpose
*sbn*B	sbnB_L	CATgTgCACgCTTTgTTgATA	qPCR
	sbnB_R	TCTTTAgCATTTTCTgTCgCAAT	qPCR
*sfa*B	sfaB_L	TTgATggAAAAggTgCAACA	qPCR
	sfaB_R	AAgCAATTAAAggCgggATT	qPCR
*fhu*D2	fhuD2_L	GAATTTgAAAgCTACTAAAgAAggACA	qPCR
	fhuD2_R	gCATgAAATCTAATgTATAAggATCg	qPCR
*ftn*A	ftnA_L	ACAgAggTgCACATgCAgAA	qPCR
	ftnA_R	AATgCATTgCTgTCATCgCC	qPCR
pgi	pgi_L	CACAAAAgCATTCgAAggTACA	qPCR
	pgi_R	TTgTggAATgTTCACTACCATgT	qPCR
*fum*C	fumC_F	ATgCTTgACCgTTgCgAAAT	qPCR
	fumC_R	AgCgCCTTCAATgTTCCATg	qPCR
